# Low-Temperature and Light Pretreatment Interactively Promote Rapid Flowering, Early Ripening, and Yield Accumulation of Winter Wheat

**DOI:** 10.3390/ijms26094280

**Published:** 2025-04-30

**Authors:** Yuanlong Wu, Runnan Shuai, Xiaoxu Zhan, Qiangui Wang, Si Tang, Tingting Gao, Yanyan Zhao, Qichang Yang, Zhonghua Bian

**Affiliations:** 1 College of Agriculture and Animal, Qinghai University, Xining 810016, China; w7474989905@163.com (Y.W.); ys220951310565@qhu.edu.cn (Q.W.); 2 Institute of Urban Agriculture, Chinese Academy of Agricultural Sciences, Chengdu 600001, China; shuairunnan@caas.cn (R.S.); zhanxiaoxu@caas.cn (X.Z.); ts970805@163.com (S.T.); gt296628685@163.com (T.G.); yangqichang@caas.cn (Q.Y.); 3 College of Agriculture and Biotechnology, Zhengzhou University, Zhengzhou 450001, China

**Keywords:** low temperature, light, flowering time, transcriptome, wheat

## Abstract

Exposing wheat (*Triticum aestivum* L.) seeds to a combination of light and low temperatures for 4–6 weeks, followed by transferring to speed breeding (SB) conditions, has been demonstrated to effectively reduce generation time in winter wheat. To reveal the underlying mechanisms of accelerated generation advancement in winter wheat, we investigated changes in transcriptome and the subsequent responses in plant growth, flowering of germinated seeds vernalized at 4 °C with white exposure (VL) or under dark conditions (VD) for 4 weeks before sowing, and subsequent growth under SB conditions. Germinated seeds without vernalization were directly sown under SB conditions and served as controls (Control). The results showed that, compared with Control and VD, VL significantly expedited vernalization, resulting in early flowering for around 6 days and accelerated ripening of progeny seeds for 13 days with a higher germination index and vigor index. The transcriptomic analysis revealed that the differently expressed genes (DEGs) involved in GA synthesis and its signal transduction both participated in the light-induced speed vernalization and the subsequent rapid growth and development of winter wheat. The MADS-box transcription factors, especially *VRN-A1* and *MADS55*, might play a vital role in the light- and low-temperature-induced early flowering. Our results stress the importance of light in vernalization and lay the groundwork for further elucidating the mechanisms underlying the light-induced speed vernalization of winter wheat.

## 1. Introduction

With the rapid increase in the world population, it is predicted that an additional 60–70% of crop production will be necessary to meet the food demands of the world’s anticipated 10 billion people by 2050 [1]. In conjunction with unpredictable adverse weather and climate change, there is an urgent need for new crop varieties with high yield and stress tolerance [2,3]. In traditional crop breeding protocols, breeding new crops requires multi-generation selection and propagation. For instance, the selection of new varieties, such as wheat and rice, generally takes approximately 10 years from initial hybridization to final commercial application [4]. Therefore, to guarantee world security, there is a pressing necessity to accelerate the process of crop breeding.

In recent years, speed breeding (SB) under controlled environments has attracted wide attention globally [5]. As early as 1980, NASA utilized continuous light to grow wheat in space, successfully accelerating wheat growth and shortening its growth period. This effort led to the development of a dwarf high-yield variety USU-Apogee with a span of less than 7 years [6,7]. Inspired by NASA, Hickey’s research group at the University of Queensland first proposed the concept of SB in a controlled environment. They successfully achieved 4–6 generations of spring wheat, barley, rape, chickpea, and other crops within one year, which afforded a new insight and provided technical support for rapid breeding [5]. Currently, the available SB protocols primarily depend on the regulation of environmental factors and exogenous substances, such as adjusting photoperiod [8], light quality [5], and plant growth regulators [9], to shorten crop cycles. However, our comprehension of the regulatory mechanisms associated with various regulatory methods or environmental factors that contribute to shortening the crop growth period remains limited.

Wheat (*Triticumaestivum* L.) is one of the most widely cultivated crops and serves as a major staple food globally, providing approximately 20% of the calories and protein consumed by humans [10,11]. Flowering signifies the transition of the crop from vegetative growth to reproductive growth, which in turn influences the duration of the crop growth period. As a long-day crop, the flowering time and flower development are mainly regulated by vernalization and photoperiod. Based on genetic differences associated with vernalization, wheat can be categorized into winter wheat and spring wheat. In winter wheat, vernalization plays a crucial role in facilitating the transition from vegetative growth to reproductive growth [12,13,14,15]. In cereal crops, the vernalization process is mainly regulated by *VRN1*, *VRN2*, *VRN3*, and *VRN4* genes [16,17]. Among them, *VRN1* is the first vernalization gene to be discovered and cloned and plays a positive regulatory role in wheat heading with its expression up-regulated in the stem tip meristem and leaves of winter wheat after low-temperature treatment [18,19]. Low-temperature treatment could dynamically recombine the *FLC* gene and chromatin, resulting in *FLC* silencing, which alleviates the inhibition of *SOC1* and *FT* expression, thereby promoting flowering [20]. It has been demonstrated that *VRN3* is capable of sensing the low-temperature treatment time and collaborated with *VRN5*, *VRN1*, and *VRN2* to inhibit *FLC* expression [21,22]. Furthermore, *VRN3* functions as a crucial hub gene in both the vernalization pathway and the photoperiodic pathway that regulates wheat flowering via interacting with *VRN1* [23].

It is well known that light serves not only as the driving force of plant photosynthesis but also functions as a crucial environmental signal that regulates plant growth and development [24], especially the flowering time of higher plants. Therefore, we hypothesize that a synergistic interaction between light and low temperature might expedite the vernalization process in winter wheat, subsequently fostering its rapid growth and development. Nevertheless, the mechanism underlying the combined effects of low temperature and light on accelerating vernalization and ultimately influencing subsequent growth and development, as well as the crop yield formation, remains elusive.

This study aimed to investigate the effects of combined low-temperature and light pretreatment of germination seeds on the flowering time, ripening, and yield accumulation of winter wheat from the physiological, crop phenotypic, and transcriptomic levels. The findings of this study could not only deepen our comprehension of the intricate interplay of multiple environmental factors in regulating the speed of vernalization of crops but also provide valuable insights and a direction for accelerating the breeding of winter crops in controlled environments.

## 2. Results

### 2.1. Combined Application of Low-Temperature and Light Pretreatment Accelerated the Growth and Yield of Winter Wheat

**Figure 1:**
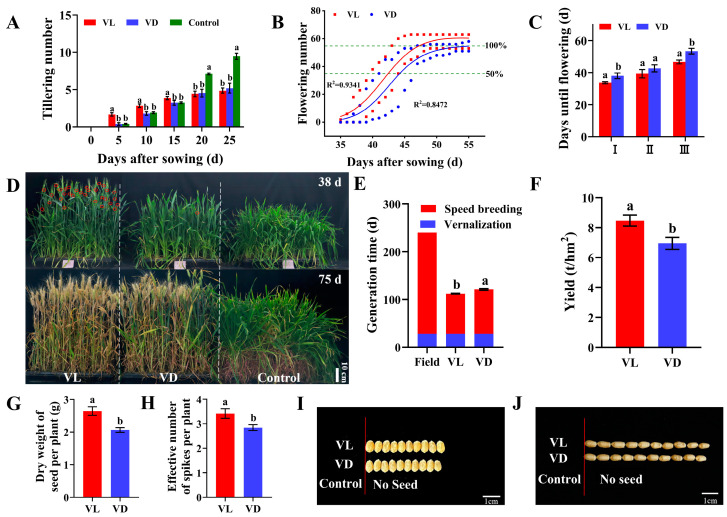
The agronomic traits of winter wheat under different light–low-temperature pretreatments (*n* = 24). (**A**) Changes in tiller number within 25 days after sowing. (**B**) Fitting analysis of flowering time under different light–low-temperature pretreatments. (**C**) The days needed for the first spike to flower (I), 50% flowering (II), and 100% flowering (III) of wheat under VL and VD treatment. (**D**) Wheat phenotypes at 38 and 75 days after sowing. (**E**) Wheat growth period under different light–low-temperature pretreatments. (**F**) Yield under different light–low-temperature coupling pretreatments. (**G**,**H**) Seed dry weight and effective number of spikes per plant in wheat under different light–low-temperature coupling pretreatments. (**I**,**J**) Wheat grain width and grain length under different light–low-temperature pretreatments. The red curve is the fitting curve for VL treatment, and the blue curve is the fitting curve for VD treatment. Different letters represent significant differences between treatments, *p* < 0.05. VL: Germinated seeds vernalized at 4 °C with light exposure for 4 weeks before sowing; VD: Germinated seeds vernalized at 4 °C in the dark for 4 weeks before sowing; Control: Germinated seeds without vernalization before sowing.

At 25 days after sowing, the plants in the Control group remained in the tillering stage of the vegetative growth period (Figure 1A). In contrast, the plants of VL and VD had completed their tillering at 20 and 25 days after sowing, respectively. Furthermore, during the initial period from day 0 to day 15, the number of tillers observed in plants under VL was significantly higher than that under both the VD and Control groups. However, no significant difference was observed in the final tiller count between VL and VD (Figure 1A). These results suggest that exposure to light during the low-temperature vernalization process of germinating seeds could significantly enhance the rapid growth of winter wheat.

To investigate the influence of light exposure during the vernalization phase of germinated seeds on the subsequent transition between growth stages in winter wheat, we performed a statistical analysis on the flowering time of each wheat plant under both VD and VL using the S-type curve of the logistic growth model (Figure 1B). The first-flowering plant of VL and VD was observed at 34.60 and 38.00 days after sowing, respectively. The duration for 50% and 100% of plants to complete flowering under VL was recorded as 38.33 and 46.67 days, respectively, whereas the corresponding durations for plants under VD were observed as 42.67 and 53.33 days. (Figure 1C).

In this study, the plants under Control demonstrated solely vegetative growth (Figure 1D) and did not initiate flowering, even after more than 150.00 days of cultivation. The harvest time for plants under VL treatment was 108.00 ± 7.30 days, which is 13 days earlier than that observed under VD treatment (121.00 ± 5.20 days). In comparison to outfield planting, which typically takes around 240 days, the growth periods from seeds to seeds for plants under VL and VD were reduced by 145.00 and 132.00 days, respectively. These results demonstrate that combined low-temperature and light pretreatment of germinated seeds not only facilitated the growth transition but also accelerated the ripening of winter wheat.

Compared with VD, the seed dry weight per plant and effective panicle number under VL increased by 28.16% and 20.23%, respectively (Figure 1G,H), while the yield significantly increased by 21.87% (Figure 1F). However, no significance was observed between VL and VD regarding other parameters, including grain length, grain width (Figure 1I,J), grouting rate, and 1000-grain weight (Appendix A).

### 2.2. Combined Low-Temperature and Light Pretreatment Improved the Germination Capacity of Progeny Seeds of Speed Breeding

**Figure 2:**
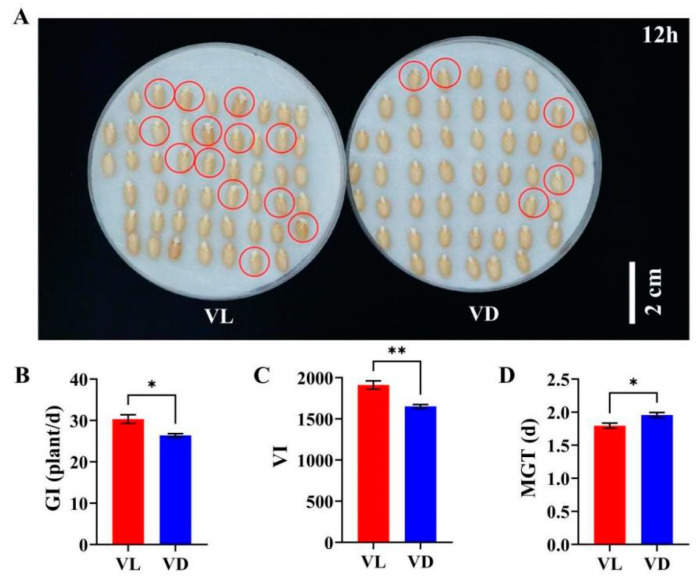
The germination capacity of progeny seeds harvested from plants under different light–low-temperature pretreatments (*n* = 24). (**A**) Seed phenotype after 12 h of germination. (**B**) Germination index (GI), (**C**) vigor index (VI), and (**D**) mean germination time (MGT) of seeds harvested under different light–low-temperature coupling pretreatments. Th red circles in this figure show germinated seeds. The * and ** in the bar charts represent the significant difference between VL and VD at *p* < 0.05 and *p* < 0.01, respectively.

To further investigate the influence of light and low-temperature pretreatment on the quality of seeds produced during the speed breeding in this study, we conducted the germination capacity assessment of progeny seeds. After 12 h germination, the number of germinated seeds harvested from under VL was higher than that from VD (Figure 2A). Compared to VD, the germination index (GI) and vigor index (VI) of seeds harvested from VL were markedly increased by 14.97% and 15.78%, respectively (Figure 2B,C). Furthermore, the MGT of VL was reduced by 9.50% relative to that of VD (Figure 2D). These results show that low-temperature and light pretreatment of germinated seeds can enhance the vitality and quality of progeny seeds in speed breeding.

### 2.3. Gas Exchange Response to Combined Low-Temperature and Light Pretreatment

**Figure 3:**
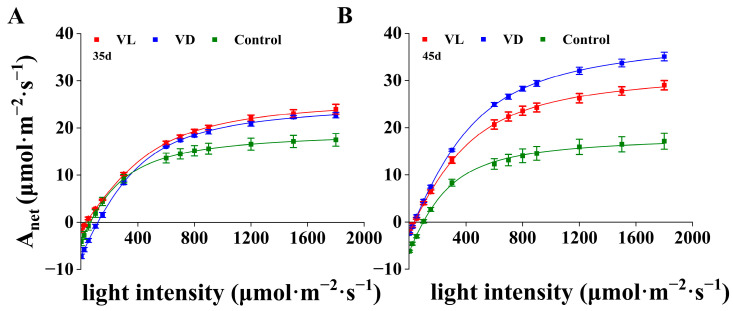
The light response curve of winter wheat under various treatments at 35 days (**A**) and 45 days (**B**) after sowing (*n* = 18).

To elucidate the effects of low-temperature and light pretreatment on photosynthetic performance at different growth stages, we measured gas exchange parameters at 35 and 45 days after sowing. At 35 days after sowing, the wheat plants of VL and VD began transitioning from the vegetative to the flowering stage, while the control group remained in the vegetative stage due to lack of vernalization. The A_net_ of wheat plants under VL and VD was significantly higher than that observed under control conditions across the established light density gradients; however, there was no significant difference between VL and VD (Figure 3A).

At 45 days after sowing, all plants under VL treatment had completed flowering, while those under VD treatment were still in the flowering stage. Compared with the light-response curves measured at 35 days, the A_net_ of wheat plants under VD and VL significantly increased at 45 days. In contrast, the values of A_net_ under Control were comparable between 35 and 45 days (Figure 3A,B). Notably, the A_net_ for VD at 45 days was higher than that observed under VL as light intensity increased from 400 to 1800 μmol m^−2^ s^−1^ (Figure 3B).

At 35 days after sowing, compared with VD, the net photosynthesis measured at growth light intensity (A_net370_) increased by 22.25% and the A_max_ increased by 15.75%, while the R_day_ decreased significantly by 77.84% when compared with the corresponding parameters under VD. Nevertheless, at 45 days, the A_net370_ and the A_max_ of plants under VD were found to be 16.22% and 21.71% higher than that under the VL group, while the R_dark_ remained significantly decreased by 60.68%. Notably, the values of Fv/Fm were comparable among Control, VL, and VD at both 35 and 45 days (Table 1).

### 2.4. Identification and Analysis of Differently Expressed Genes

**Figure 4:**
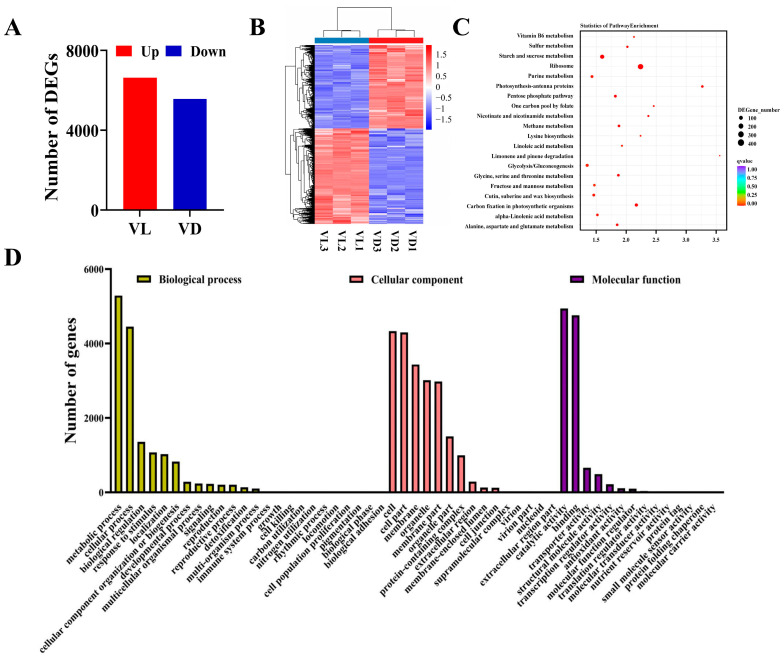
The transcriptomic response of germinated winter wheat seeds subjected to VL and VD. (**A**) Number of differentially expressed genes (DEGs) in different light–low-temperature pretreatments. (**B**) Hierarchical clustering analysis of the expression pattern of the DEGs. (**C**) The top 20 KEGG pathways for the prominent enriched differentially expressed genes in the different light–low-temperature pretreatments. (**D**) Functional classification of DEG responses to different light–low-temperature pretreatments.

To obtain insight into the transcript regulation under different light–low-temperature pretreatments on germinated seeds, we conducted a transcriptome analysis on germinated seeds exposed to low temperatures with or without light for four weeks. A total of 11,931 DEGs were identified in the comparison of VL and VD, with 6366 up-regulated and 5565 down-regulated (Figure 4A). Furthermore, a hierarchical clustering analysis was performed to provide an overview of the expression pattern of DEGs (Figure 4B). Most of the genes with lower expression in VD species were expressed at higher levels in VL species, and vice versa, indicating that germinated seeds undergo complex changes in transcript levels after combined low-temperature and light pretreatment.

**Figure A2:**
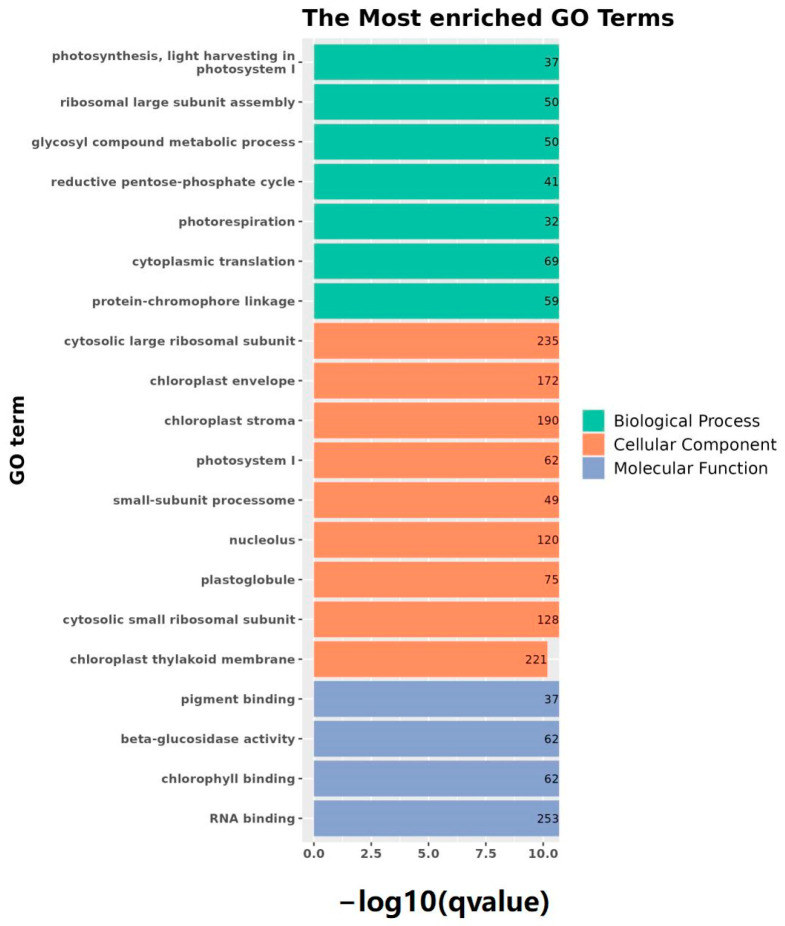
Analysis of GO enrichment under different light–low-temperature coupling pretreatments.

To reveal the light exposure-induced transcriptomes during the low-temperature vernalization of germinated seeds, we carried out a GO enrichment analysis to classify the DEGs of VD vs. VL. Compared with VD, a total of 9352 DEGs in VL were markedly enriched in the cellular component, biological process, and molecular function, the three main GO categories (Appendix A, Figure A2 and Figure 4D). The GO terms markedly enriched in the biological process categories include ‘metabolic process’, ‘cellular process’, ‘biological regulation’, ‘response to stimulus’, ‘reproduction’, and ‘reproductive process’. In the cellular component category, the significantly enriched GO terms were observed in ‘cell’, ‘cell part’, ‘membrane’, ‘membrane part’, and ‘protein-containing complex’. Furthermore, ‘binding’, ‘catalytic activity’, ‘transporter activity’, ‘transporter regulator activity’, and ‘structural function regulator’ in the cellular component were markedly enriched in the comparison of VL vs. VD (Figure 4C).

To further investigate the biological functions of DEGs, a pathway-enriched analysis was conducted based on the KEGG database. In the VD vs. VL comparison, a total of 3609 DEGs were annotated to 122 metabolic pathways, and the top 20 DEGs that were significantly enriched are presented in Figure 4C. The DEGs were predominantly enriched in ‘limonene and pinene degradation’, ‘photosynthesis-antenna proteins’, ‘one carbon pool by folate’, ‘nicotinate and nicotinamide metabolism’, and ‘ribosome’, and each metabolic pathway contained 11, 51, 18, 25, and 428 DEGs, accounting for 0.30%, 1.41%, 0.50%, 0.69%, and 11.86% of the total DEGs, respectively (Figure 4C).

### 2.5. Expression Profiles of Flowering-Related Gene Response to Low Temperature and Different Light Conditions

**Figure 5:**
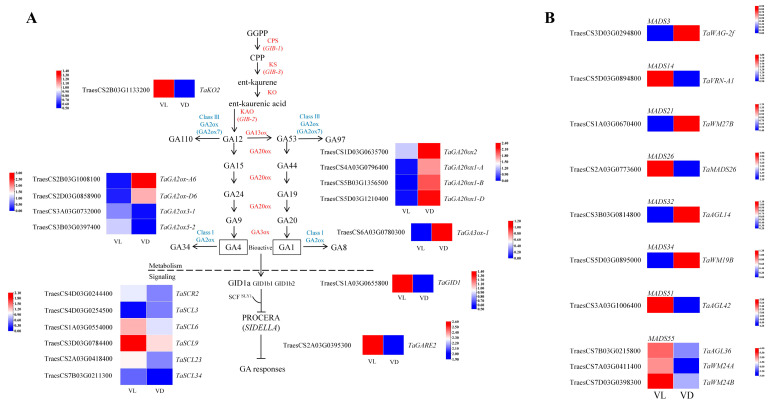
Heatmap of relative expression profiles of DEGs annotated in gibberellin pathway and flowering of MADS-box under VL and VD. (**A**) Heatmap of relative expression of DEGs in the gibberellin pathway-related structural genes in response to VL and VD. (**B**) Heatmap of relative expression of DEGs annotated in MADS-box family VL and VD.

In this study, combined low-temperature and light exposure during the vernalization of germinated seeds led to earlier heading or flowering than other treatments (Figure 1B,C). To investigate the effects of light exposure during vernalization on the regulation of flowering in winter wheat, we present the DEGs involved in the regulation of flowering in Figure 5, which included the members from the MADS-box family and gibberellin (GA) biosynthesis and signaling transduction, based on the DEGs identified through KEGG pathway analysis.

Compared to VD, VD up-regulated *TaKO2* (TraesCS2b03g1133200), which encodes the key enzyme KO that catalyzes GA precursor synthesis. In contrast, VL led to the down-regulation of several genes involved in GA biosynthesis, including *TaGA20ox1-A* (TraesCS4A03G0796400), *TaGA20ox1-B* (TraesCs5B03G1356500), *TaGA20ox1-C* (TraesCS5D03G1210400), *TaGA20ox2* (TraesCS1D03G0635700), and *TaGA3ox-1* (TraesCS6A03G0780300). Additionally, when compared to VD, VL led to the up-regulation of genes associated with deactivating enzymes, specifically TaGA2ox3-1 (TraesCS3A03G0732000) and *TaGA2ox5-2* (TraesCS3B03G0397400) (Figure 5A). Regarding the GA signaling transduction pathway, VL was found to up-regulate *TaGID1* (TraesCS1A03G0655800); however, it also caused a significant down-regulation of most differently expressed genes encoding DELLA proteins (Figure 5A).

In higher plants, the MADS-box gene family is one of the most critical gene families involved in the regulation of flowering. In our current study, we identified ten DEGs belonging to the MADS-box gene family that are implicated in flowering regulation. Among these, six genes were found to be up-regulated, while four genes were down-regulated (Figure 5B). Specifically, *TaVRN-A1* (TraesCS5D03G0894800), a key gene associated with vernalization, exhibited up-regulation under VL conditions compared to VD conditions. Furthermore, *TaMADS26* (TraesCS2A03G0773600), *TaAGL42* (TraesCS3A03G1006400), and three DEGs belonging to MADS55 (TraesCS7B03G0215800, *TaAGL36*; TraesCS7A03G0411400, *TaWM24A*; TraesCS7D03G0398300, *TaWM24B*) were all up-regulated under VL when compared with VD (Figure 5B).

### 2.6. Validation of Differently Expressed Genes by qPCR

**Figure A3:**
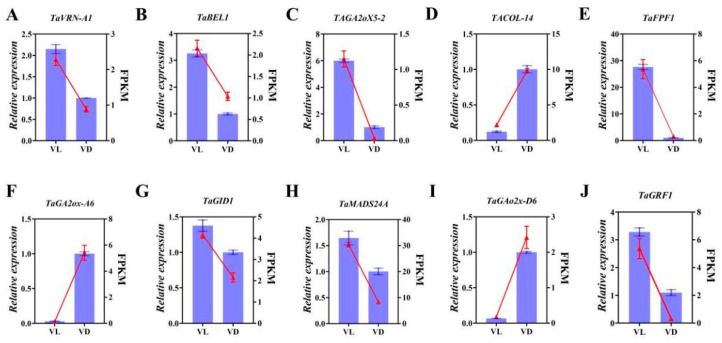
The expression patterns of 10 randomly selected DEGs by RNA-Seq and qPCR. Red line represents the transcriptome expression abundance of selected DEGS.

To validate the accuracy of the RNA-Seq data, 10 DEGs were randomly selected, and their expression profiles were verified by qRT-PCR (Appendix A, Figure A3). The qPCR results indicated that the relative transcription profiles of these selected DEGs under various treatments closely resembled the FPKM expression patterns observed in the RNA-Seq data. These results demonstrated the accuracy and reliability of the RNA-Seq results.

## 3. Discussion

Vernalization is one of the most critical stages in regulating the flowering and yield accumulation of winter cereal crops. Accelerating the vernalization process is an effective strategy for shortening the growth period and facilitating speed breeding of winter cereal crops [25]. To our knowledge, this study is the first to systematically demonstrate that light exposure can accelerate the vernalization process in germinated winter wheat seeds, subsequently exerting a positive influence on the yield and germination capacity of progeny seed in speed breeding.

### 3.1. Low-Temperature and Light Exposure Speed up the Vernalization of Germinated Seeds and Promote Rapid Growth in Winter Wheat

Flowering serves as a critical indicator of the transition from vegetative to reproductive growth, with the timing of flowering being crucial for successful reproduction and optimal seed production throughout the plant growth period [26,27]. Previous studies have demonstrated that the flowering of winter crops can be accelerated by prolonged exposure to cold temperatures [25,28]. A few weeks of cold exposure are generally sufficient to induce flowering; however, prolonged periods of vernalization can further enhance the flowering process, up to a point where the response reaches saturation [25]. This enhancement in flowering may require more than 6 weeks of vernalization [28]. Shourbalal et al. (2019) demonstrated that the cold stratification of germinated winter wheat seeds effectively shortened the vernalization period in winter wheat [29]. In this study, compared to VD, plants subjected to VL completed tillering and flowering at an earlier stage (Figure 1A–C), suggesting that light exposure during the low-temperature treatment of germinating seeds can accelerate the vernalization process of winter wheat. In other words, light could partially substitute for vernalization, thereby reducing the duration of the vernalization period.

The vegetative growth phase of crops, especially winter cereals, typically represents the predominant portion of the overall growth duration. Promoting the early flowering of crops is an effective strategy for significantly shortening their growth period [30]. The flowering and early ripening of crops are both influenced by a variety of factors, including genotype, environmental conditions, and stress. In this study, the comparable values of Fv/Fm across the treatments suggest that the speed breeding environments and the pretreatment of germinated seeds used in this study did not impose adverse effects or stress on the growth and development of winter plants (Table 1). Together with the enhanced photosynthetic capacity and the resulting higher final yield observed under VL compared to VD (Figure 3 and Table 1), these results demonstrated that light exposure during low-temperature vernalization positively affects the subsequently rapid growth, flowering, and repining of winter wheat. However, the precise mechanisms underlying this positive subsequent regulatory response remain unclear.

### 3.2. Low-Temperature and Light Pretreatment Enhances the Vitality and Quality of Progeny Seeds in Speed Breeding

In crop breeding, the vitality and quality of progeny seeds are crucial agronomic indicators that breeders prioritize, as they play a vital role in the successful selection of new varieties [31]. In the current existing speed breeding protocols, early harvesting is a commonly employed strategy to shorten the crop breeding generation cycle [32,33]. However, this approach often comes at the expense of seed vitality and yield, which adversely affects the production of sufficient parental lines during the breeding process. Therefore, enhancing seed vitality and quantity in rapid breeding is crucial for establishing an effective rapid breeding technology system. To eliminate the influence of other factors on seed quality assessment, we used naturally matured seeds from speed breeding to investigate how light exposure and low-temperature pretreatments affect progeny seed germination capacity. The higher vitality and yield of progeny seeds harvested from VL compared with VD treatment (Figure 1 and Figure 2) demonstrated the beneficial effects of light treatment during low-temperature vernalization in enhancing the vitality and quality of progeny seeds in speed breeding. Therefore, if this approach is combined with the harvesting of premature seeds, it may further reduce the generation time of winter wheat without compromising seed vitality [5].

### 3.3. The Molecular Basis of Speed Vernalization Under Light and Low-Temperature Pretreatment

It has long been known that tiller number is one of the important factors determining cereal plant architecture and grain yield [34,35]. The development of tillers is governed by a complex network involving genetic factors, plant hormones, and environmental factors. Gibberellins (GAs), as important phytohormones, are known to inhibit tillering [34,36]. The application of paclobutrazol, an inhibitor of GA biosynthesis, has been found to promote the initiation of wheat tillering [37]. Recently, remarkable evidence has elucidated the mechanism underlying GAs inhibiting the tillering of cereals, especially in rice [34]. It was demonstrated that DELLA proteins, the central repressors of GA signaling, interact with MOC1 to protect MOC1 from degradation, thereby promoting the growth and development of rice tiller buds [34]. In this study, VL inhibited the expression of key genes related to GA synthesis (e.g., *TaGA20ox1s*, *TaGA20ox2*, and *TaGA3ox-1*) and up-regulated the expression of DELLA-related genes (Figure 5). Therefore, the accelerated tillering and rapid completion of the tillering process of winter wheat (Figure 1A) could be partially attributed to the fact that, during the low-temperature vernalization of germinated seeds, light exposure releases GA and its signaling transduction-induced inhibition to the onset of tillering, thereby promoting the rapid growth and tillering of winter wheat [37].

Until now, the molecular basis of vernalization-induced flowering in cereals has been extensively studied [28]. Genetic analyses in barley and wheat have identified *VRN1*, *VRN2*, and *VRN3* as the core genes determining the vernalization requirement [23,28]. Vernalization accelerates the initiation of inflorescence by inducing the *VRN1* gene [38], encoding an APETALA1-like MADS-box transcription factor (TF) that promotes inflorescence meristem identity [39]. In varieties that necessitate vernalization for flowering, the extent to which *VRN1* expression is induced correlates with the duration of vernalization exposure [40], resulting in a quantitative effect on the timing of inflorescence initiation. Therefore, when compared with VD, the up-regulated *TaVRNA1* of germinated seeds (Figure 5B) and subsequent early flowering under VL (Figure 1B,C) further highlight the positive impact of light exposure on accelerating winter wheat vernalization and speed breeding.

**Figure 6:**
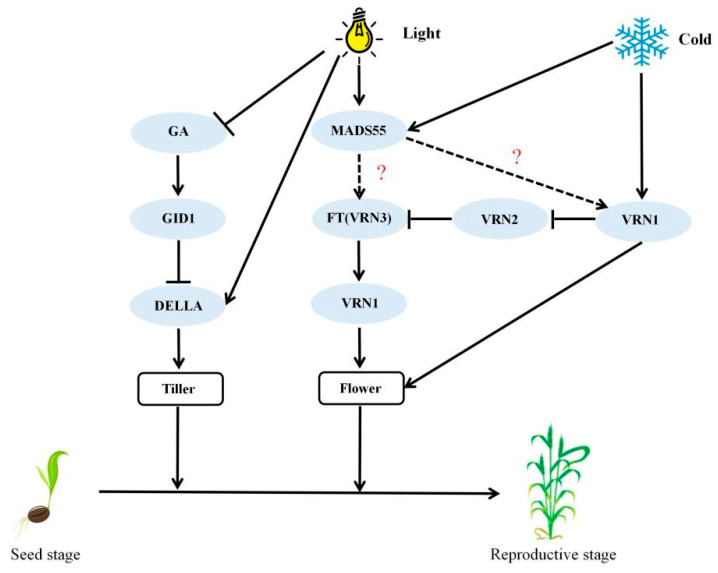
Prediction of the rapid flowering model of wheat under different light–low-temperature pretreatments. → It has been shown to have a facilitating effect. ⊣ It has been shown to have an inhibitory effect. ? and ⇢ It is speculated that it may have a facilitating effect.

It has been proven that MADS-box TFs usually work by forming hetero- or homo-dimers [41]. In Polish wheat (*Triticum polonicum*), *MADS55* is encoded by VRT-A2, which is an ortholog of wheat VRT2 [42]. The MADS-box gene *VEGETATIVE TO REPRODUCTIVE TRANSITION 2* (*VRT2*) cooperates with *TaVRN1* to regulate vernalization-induced flowering in wheat [43]. In this study, the DEGs encoded MADS55 were all found to be up regulated in the comparison of VL vs. VD (Figure 5B), indicating that related genes of MADS55 (*TaMADS55s*) may be involved in the light- and low-temperature-regulated flowering of winter wheat via directly or indirectly interacting with vernalization genes such as *TaVRN1* and *TaVRN3* (Figure 6). However, ongoing studies are essential to elucidate the regulatory mechanisms of *VRN-A1* and *MADS55* with light-induced speed vernalization and the subsequent acceleration of generation advancement in winter wheat.

## 4. Materials and Methods

### 4.1. Experimental Materials and Growth Conditions

**Figure 7:**
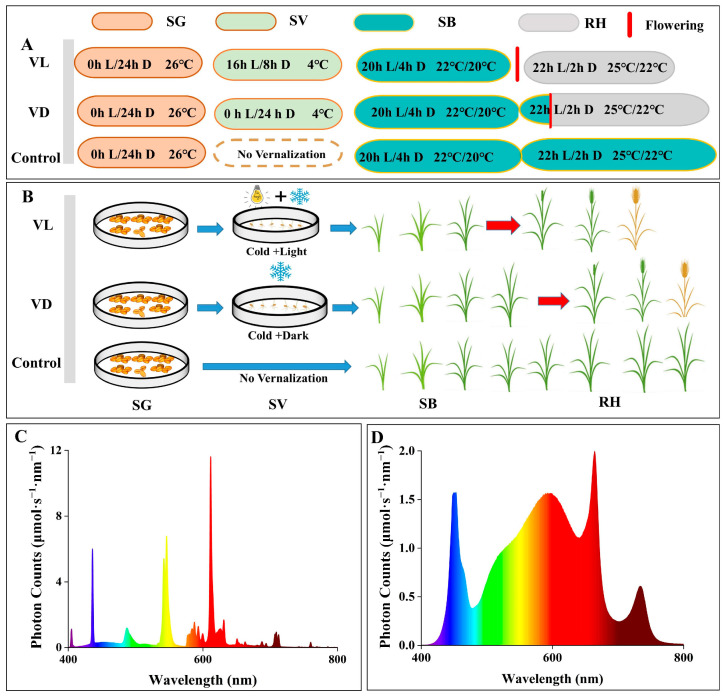
Experiment design and growth conditions for the whole growth period of winter wheat. (**A**) The schematic diagram illustrates the experimental design and delineates the nomenclature of the various stages of plant growth under VL, VD, and Control. (**B**) The photoperiod and temperature conditions of plants at various growth stages. (**C**) the light spectra of the light sources utilized during the SV stage under VL treatment. (**D**) light spectra of the light sources utilized during the stages of SB and RH in this study. Different colors in C and D represent light spectra of light sources.

The seeds of winter wheat (*Triticum aestivum* L. vs. Lu mai 27) were sterilized with a 5% hydrogen peroxide solution for 5 min, followed by washing with distilled water before germination. For the germination process, these seeds were placed in petri dishes with moist filter paper and incubated at 26 °C for 48 h. The healthy and germinated seeds were selected, randomly divided into two groups, and placed in sprout trays containing wet gauze affixed to the bottom. One group of germinated seeds was vernalized at 4 °C under dark (0 μmol m^−2^ s^−1^) conditions (VD) for four weeks (Figure 7A,B), while the other group of germinated seeds was vernalized at 4 °C and concomitantly exposed to a 180 ± 10 μmol m^−2^ s^−1^ white light (VL) with a 16/8 h photoperiod for four weeks (Figure 7A–C). During the vernalization and different light treatments, the CO_2_ level and air relative humidity were set at 400 ppm and 70%, respectively. Additionally, the germinated seeds were irrigated with tap water every other day to fulfill their moisture requirements.

After four weeks (28 days) of combined vernalization and different light condition treatments, similarly sized and healthy germinated seeds of VL and VD were sown into plastic seedling trays with substrate cultivation (*v*:*v*, peat: vermiculite = 3:1). Another group of germinated seeds without vernalization treatment was sown in the same substrate as VL and VD, and those plants are referred to as Control. The planting density for all three treatments was set at 229 plants/m^2^, and these were grown under sunlight white-light LEDs (Figure 7D) in an environmentally controlled growth chamber. The light intensity, day/night temperature, photoperiod, CO_2_ level, and relative humidity in the growth chamber were set at 370 ± 10 μmol m^−2^ s^−1^, 22 ± 1/20 ± 1 °C, 20 h, 400 ppm, and 60 ± 5%, respectively.

Fresh-prepared Hoagland nutrient solution (pH = 6.1 ± 0.5, EC = 2.1 ± 0.3 ms/cm) was added from the bottom to supply nutrition for wheat plants with an interval of 5 days. When vernalized wheat plants entered the grain filling stage, the day and night temperature and photoperiod were changed to 25 ± 1/22 ± 1 °C and 22 h, respectively, to accelerate the seed ripening of wheat. This experiment was independently repeated three times. The experiment design and growth conditions at various growth stages throughout this study are presented in Figure 7.

### 4.2. Morphological Indicators and Yield Determination

The tillering number of wheat plants was recorded at 5-day intervals after sowing. The days required for wheat flowering under each treatment were recorded and subsequently fitted with a logistic growth model [44]. At the end of the maturity stage, the yield and agronomy parameters were calculated, including seed dry weight per plant, effective panicle number, and filling rate. This experiment was independently repeated three times, with eight replications per treatment in each independent experiment (n = 24).

### 4.3. Gas Exchange Parameter Determination

At 35 and 45 days after sowing, the second youngest and fully expanded leaves of wheat were used for gas exchange parameter measurements. The net photosynthesis rate (A_net_) and chlorophyll a fluorescence parameter were concomitantly determined by a LI-6800 Portable Photosynthesis System (LI-6800F, LI-COR, Inc, Lincoln, NE, USA). Before the measurement determining the maximum quantum efficiency of the dark period (Fv/Fm), the leaves were dark-adapted for 30 min. The light response curve of A_net_ (A-Q curve) was measured using the method described by Bian et al. (2021) [45]. Briefly, the light intensities of the A-Q curve were set as follows: 1800, 1500, 1200, 900, 800, 700, 600, 300, 150, 100, 50, 20, and 0 μmol m^−2^ s^−1^ PPFD. During the A-Q curve measurement, the actinic light in the leaf chamber was provided by red and blue LED light sources (90% red, 10% blue). The CO_2_ level, air temperature, relative humidity, and airflow rate in the leaf chamber were set at 400 ppm, 25 °C, 65%, and 500 μmol s^−1^, respectively. The A_net_ obtained at the growth light intensity was used to estimate the photosynthesis performance. The method of Thornley was used for A-Q curve fitting [46], while the responses of chlorophyll a fluorescence parameters to the changes of PPFD were calculated as described by Baker (2008) [47]. The gas exchange measurement was independently repeated three times, with six replications per treatment in each independent experiment (n = 18).

### 4.4. Determination of Germination Capacity of Progeny Seeds

The progeny seeds were harvested after natural maturity. The harvested seeds with uniform maturity, intact grains, and uniform size were selected and sterilized by soaking in 5% H_2_O_2_ for 5 min. The seeds were then rinsed with distilled water 5 times and placed neatly in a germination box before incubating at 26 °C for 72 h. The number of germinated seeds was recorded at 12 h intervals. After incubating for 72 h, 20 seeds of each treatment with uniform growth were randomly selected for the root length and the number of adventitious roots of measurement. The method of Ruiying Shi (2022) [48] was used to calculate the germination rate, germination potential, mean germination time (MGT), germination index (GI), and vigor index (VI), as follows:

(1) $$MGT=\frac{G_{1}T_{1}+G_{2}T_{2}+\ldots +G_{n}T_{n}}{G_{1}+G_{2}+\ldots +G_{n}}$$

(2) $$GI=\sum \frac{G_{t}}{D_{t}}$$

(3) VI=S×GI

where G_n_ is the number of germinated seeds (grains) corresponding to the number of germinating days, Tn is the number of germinating days (d), G_t_ is the number of germinated seeds (grains) corresponding to the time t, D_t_ is the number of germinating days (d), and S is the average root length of seeds (cm).

### 4.5. RNA Extraction and Transcriptome Sequencing

At the end of four weeks of vernalization, the sprouts of germinated seeds under VL and VD were sampled for total RNA extraction. The total RNA was extracted using an RNA Easy Fast Plant Tissue RNA Rapid Extraction Kit (Tiangen, Beijing, China). Total RNA concentration and purity were determined using a NanoDrop 2000C spectrophotometer (Thermo Scientific, Waltham, MA, USA), with purity assessed by OD260/280 and OD260/230 values. The RNA integrity was tested using an Agilent 2100 system (Agilent Technologies, Santa Clara, CA, USA) for RIN values, 28S/18S ratios, uplift of the baseline, and 5S peaks.

The total RNA extracted from the samples was utilized for RNA-Seq library construction and subsequent sequencing (Beijing Group Biotechnology Co., Ltd., Beijing, China). Specifically, magnetic beads with Oligo (dT) were employed to enrich eukaryotic mRNAs by binding to the ployA tail of the mRNA through A-T complementary pairing. Following this, mRNA was fragmented into short segments by adding a fragmentation buffer. Single-stranded cDNA was synthesized using six-base random primers (random hexamers), with mRNA serving as a template. Subsequently, double-stranded cDNA was synthesized with the addition of buffer, dNTPs, and DNA polymerase I. The resulting double-stranded cDNA was purified using AMPure XP beads. The purified double-stranded cDNA was then end-repaired, A-tailed, and ligated to sequencing junctions, followed by fragment size selection with AMPure XP beads. PCR enrichment was performed to obtain the final cDNA library. Finally, the enriched cDNA libraries were assessed for library quality using an Agilent 2100 system prior to sequencing via synthesis (SBS) technology based on the Illumina HiSeq high-throughput sequencing platform.

### 4.6. Transcriptomic Analysis

Clean reads were generated by initially eliminating reads that contained junctions, followed by the removal of low-quality reads. This process includes the exclusion of reads in which the proportion of N bases removed exceeded 10%, as well as those where more than 50% of the total read consisted of bases with a quality value of Q ≤ 10. The clean reads were compared and positionally analyzed with the published genome sequence sketches of Chinese spring wheat (the bread wheat cultivar Chinese Spring, IWGSC) using Hisat2 [49]. This analysis provided positional information regarding reference genomes or genes, as well as specific sequence feature information pertinent to the sequenced samples. The expression levels of each gene across different treatments were quantified using htseq-count software(V0.6.0, EMBL Heidelberg, Germany) and the FPKM (Fragments Per Kilobase of transcript per Million fragments mapped) method. Subsequently, differences in gene expression between treatments were assessed utilizing DESeq2 (V1.10.1, Love MI et al., University of North Carolina, USA) software, with |Fold Change| ≥ 2.0 and FDR < 0.01 established as screening criteria. The differently expressed genes identified were subjected to Gene Ontology (GO) functional enrichment analysis and Kyoto Encyclopedia of Genes and Genomes (KEGG) pathway enrichment analysis, with *p*-value < 0.05 classified as significant enrichment.

### 4.7. Validation of RNA-Seq Data by qRT-PCR

Ten DEGs were randomly selected to validate the accuracy of RNA-Seq data by qRT-PCR. The primers for those selected DEGs were designed using PrimerPremier6.0 (Premier Biosoft International, Palo Alto, CA, USA). The TaActin1 from wheat was utilized as the internal reference gene [50]. The details of these selected genes, along with their corresponding primer sequences, are summarized in Appendix A, Table A1. Total RNA, which was used for the RNA-Seq sequence, was used for cDNA synthesis. The qRT-PCR was performed on a Bio-Rad CFX96™ Touch (Bio-Rad Laboratories Inc., Hercules, CA, USA) using an SsoFast Eva Green Supermix kit (Bio-Rad Laboratories Inc., USA). The reaction condition was set to the first step, high-temperature denaturation (95 °C, 30 s). In the second step, annealing and amplification (95 °C, 5 s and 57 °C, 5 s) were performed 40 times. Real-time qPCR was repeated three times based on the three separate RNA extracts from three samples (six plants per sample, three samples per treatment). The relative expressions of those selected genes were calculated by the 2^−ΔΔCt^ method, as described by Livak and Schmittgen (2001) [51].

### 4.8. Data Analysis

In this study, all measurements were repeated three times independently, with the data collected from 18 to 24 plants. All the data were analyzed using SPSS (V27.0, IBM Corp., Armonk, NY, USA). For the significant difference analysis of tiller number, one-way analysis of variance (ANOVA) and Duncan multiple range test at *p* < 0.05 were used. Paired sample T-test and Fisher’s protected LSD at *p* < 0.05 were utilized for all other comparisons. Photosynthetic parameter fitting was performed in Origin 2023 (OriginLab Corporation, Northampton, MA, USA) and Prism (V10.0, GraphPad Software Inc., San Diego, CA, USA).

## 5. Conclusions

This study confirmed that light exposure during the vernalization of germinated seeds has a positive subsequent function on the speed breeding of winter wheat via accelerating flowering, early ripening, and enhancing the quality of progeny seeds. According to the transcriptomic analysis, the identified key genes encoding GA synthesis and its signal transduction indicate the involvement of the GA pathway in the light-induced speed vernalization and subsequently rapid growth and development of winter wheat. The response of MADS-box transcription factors, especially *VRN-A1* and *MADS55*, to light- and low-temperature-induced speed vernalization were also identified. The transcriptomic data provide a foundation for elucidating the mechanisms of light-induced speed vernalization in germinated winter wheat seeds under low temperatures.

## Figures and Tables

**Table 1:** The fitting and measured parameters of gas exchange under various treatments (n = 18).

Days After Sowing	Treatment	Measured		Fitting		
		A_net370_ (umol m^−2^·s^−1^)	Fv/Fm	A_max_ (umol m^−2^·s^−1^)	AQE	R_dark_ (umol·m^−2^·s^−1^)
35 d	Control	9.40 ± 0.38 b	0.818 ± 0.02 a	23.60 ± 0.16 c	0.07± 0.01 a	4.11 ±0.05 b
	VL	10.22 ± 0.62 a	0.835 ± 0.01 a	32.92 ± 0.35 a	0.05 ± 0.01 a	1.58 ± 0.06 c
	VD	8.36 ± 0.70 c	0.826 ± 0.01 a	28.44 ± 0.56 b	0.07 ± 0.01 a	7.13 ± 0.13 a
45 d	Control	8.89 ± 1.57 c	0.834 ± 0.02 a	24.69 ± 0.80 c	0.07 ± 0.01 a	6.18 ±0.09 a
	VL	13.13 ± 1.61 b	0.829 ± 0.02 a	34.77 ± 0.91 b	0.06 ± 0.01 a	2.18 ± 0.12 b
	VD	15.26 ± 0.58 a	0.839 ± 0.01 a	42.32 ± 0.35 a	0.07 ± 0.01 a	2.43 ± 0.04 b

Different lowercase letters in the same column represent significant differences between treatments at *p* < 0.05.

**Figure A1:**
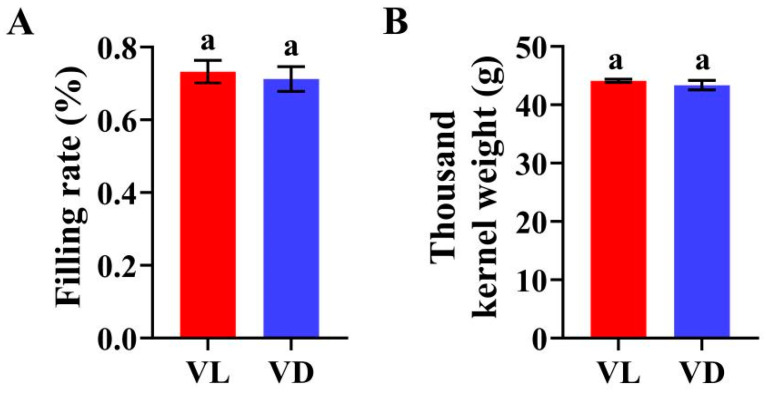
Factors affecting the yield composition of winter wheat under different light–low-temperature coupling pretreatments. (**A**) Filling rate of wheat under different light–low-temperature coupling pretreatments. (**B**) Thousand kernel weight of wheat under different light–low-temperature coupling pretreatments. Different letters represent significant differences between treatments, *p* < 0.05.

## Data Availability

The original contributions presented in the study are publicly available. These data can be found here: NCBI SRA database BioProject accession number PRJNA1220346 https://www.ncbi.nlm.nih.gov/), “URL (2025-02-10)”.

## Abbreviations

The following abbreviations are used in this manuscript:

SV	Speed vernalization
SB	Speed breeding
VL	Vernalization with light
VD	Vernalization with dark
Control	No vernalization treatment
SG	Seed germinate
RH	Ripening and harvest

## Appendix A

**Table A1:** The details of primers used for qRT-PCR validation.

Gene Name	Forward Primer (5′-3′)	Reverse Primer (5′-3′)
*VRN-A1*	GGCGCAGCAAGATCAAACTC	ATGTGGCTCACCATCCACG
*BEL1*	GGCTATGGAAGCCCATGATA	GAAGCAGTCACCAGGGTTGT
*GA2ox5-2*	GAGAACTAGCCAGCGACCAG	CGACAAAGAGGCCGAGATAG
*COL14*	ACGACGACGAAGAGCAACTT	TTCAGCAGCTGCACCATATC
*FPF1*	CAGCCAGCTAGAGAAGGGATG	CGATGTCGCGGTTCTCGTAG
*GA2ox-A6*	AAGAGCGTGGAGCACAAAGT	GTGTGATTCATGCCGTCAAC
*GID1*	CCACCATCGGCTTCTACCTGCTGTC	GGCGAGCTCATCCACGACGAGAC
*MADS24A*	AGCAGCCGGCTATTGATTTA	CCCTTGTGTTGGAGGTCACT
*GA2ox-D6*	ACAGCGACTTCCTCACCATC	ACTTTGTGCTCCACGCTCTT
*GRF1*	TACGCCATCGACCTGTCCAGC	ATGGAGATGGAGAGCTGCGTC
*Actin*	GTTGGTGATGAGGCCCAATC	GTGCTACACGGAGCTCATTG
